# Medical and Surgical Report of the Presbyterian Hospital in the City of New York

**Published:** 1901-01

**Authors:** 


### Books and Magazines.

---

MEDICAL AND SURGICAL REPORT OF THE PRESBYTERIAN HOSPITAL IN THE CITY OF NEW YORK. Vol. IV. January, 1900. Edited by Andrew J McCash, M. D., W. Gilman Thompson, M. D. Pp. 223. Trow Directory Printing and Bookbinding Co., New York.

This report consists of a review of the work of the hospital for the year. It classifies the cases and gives statistics showing the results of the treatment. The book contains many valuable articles and suggestions. T. J. B.

